# SET contributes to the epithelial-mesenchymal transition of pancreatic cancer

**DOI:** 10.18632/oncotarget.19067

**Published:** 2017-07-07

**Authors:** Hardik R. Mody, Sau Wai Hung, Kineta Naidu, Haesung Lee, Caitlin A. Gilbert, Toan Thanh Hoang, Rakesh K. Pathak, Radhika Manoharan, Shanmugam Muruganandan, Rajgopal Govindarajan

**Affiliations:** ^1^ The Comprehensive Cancer Center, The Ohio State University, Columbus, OH, USA; ^2^ Division of Pharmaceutics and Pharmaceutical Chemistry, The Ohio State University, Columbus, OH, USA; ^3^ Department of Pharmaceutical and Biomedical Sciences, The University of Georgia, Athens, GA, USA; ^4^ Department of Biochemistry and Molecular Biology, The University of Georgia, Athens, GA, USA

**Keywords:** SET, EMT, pancreatic cancer, N-cadherin, signaling

## Abstract

Pancreatic cancer has a devastating prognosis due to 80-90% of diagnostic cases occurring when metastasis has already presented. Activation of the epithelial-mesenchymal transition (EMT) is a prerequisite for metastasis because it allows for the dissemination of tumor cells to blood stream and secondary organs. Here, we sought to determine the role of SET oncoprotein, an endogenous inhibitor of PP2A, in EMT and pancreatic tumor progression. Among the two major isoforms of SET (isoform 1 and isoform 2), higher protein levels of SET isoform 2 were identified in aggressive pancreatic cancer cell lines. Overexpressing SET isoform 2, and to a lesser extent SET isoform 1, in epithelial cell lines promoted EMT-like features by inducing mesenchymal characteristics and promoting cellular proliferation, migration, invasion, and colony formation. Consistently, knockdown of SET isoforms in the mesenchymal cell line partially resisted these characteristics and promoted epithelial features. SET-induced EMT was likely facilitated by increased N-cadherin overexpression, decreased PP2A activity and/or increased expression of key EMT-driving transcription factors. Additionally, SET overexpression activated the Rac1/JNK/c-Jun signaling pathway that induced transcriptional activation of N-cadherin expression. *In vivo*, SET isoform 2 overexpression significantly correlated with increased N-cadherin in human PDAC and to tumor burden and metastatic ability in an orthotopic mouse tumor model. These findings identify a new role for SET in cancer and have implications for the design and targeting of SET for intervening pancreatic tumor progression.

## INTRODUCTION

One of the key reasons for why pancreatic cancer has a poor prognosis is due to 80-90% of diagnostic cases occurring at stage IV, when metastasis has already occurred and the median survival is only 4.5 months [1]. Metastasis is an early event in pancreatic cancer and occurs after cells have undergone EMT. Activation of EMT allows for the dissemination of tumor cells; hence, EMT is considered a prerequisite for metastasis [2]. EMT is well-characterized at the molecular level during which cells transform from a cuboidal shape with polarity to a squamous shape lacking polarity. In addition to reprogramming of numerous genes, a switch from epithelial (E)-cadherin to neuronal (N)-cadherin called cadherin switching is also evident in EMT. This transition allows primary tumor cells to migrate and invade into the bloodstream and promote metastatic tumor formation.

**Figure 1:**
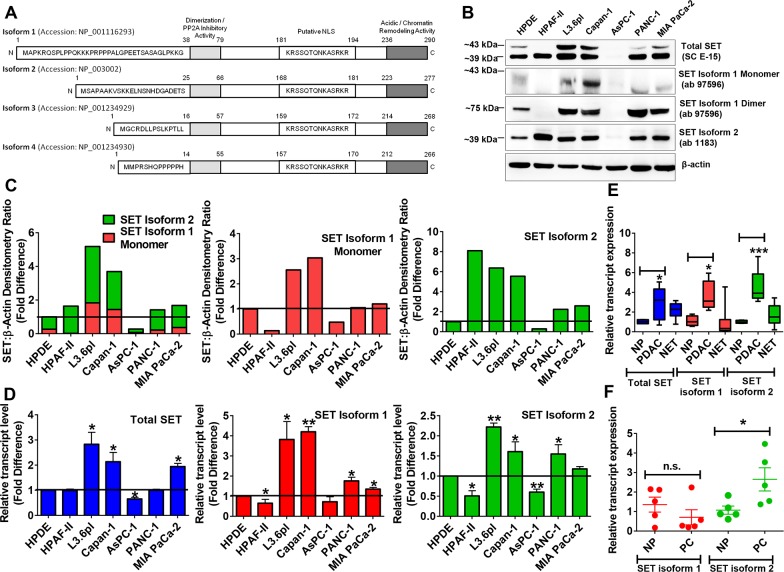
SET is frequently overexpressed in pancreatic cancer **(A)** Schematic representation of 4 SET isoforms. Isoforms 1 and 2 differ only in the first 37 (isoform 1) and 24 (isoform 2) amino acids of the protein. **(B and C)** Protein levels of total SET as well as SET isoform 2 were significantly increased in all pancreatic cancerous cell lines except AsPC-1. SET isoform 1 (especially SET Isoform 1 Dimer) showed significant increases except for AsPC-1 & HPAF-II. Whole cell lysates (50 μg) prepared with TNE (Tris-NP40-EDTA) Lysis Buffer were subjected to Western blotting analysis. β-actin, the internal loading control, is shown with a representative blot. The bands were quantified by densitometry and SET:β-actin ratios plotted (C). **(D)** SET transcript levels were significantly increased in a subset of pancreatic cancer cell lines compared with HPDE similar to Western blotting results. **(E)** All SET transcripts (total, isoform 1 and isoform 2) are overexpressed in PDAC but not in NET tissue samples. NP, normal pancreas (n=4). PDAC, pancreatic ductal adenocarcinoma (n=5). NET, neuroendocrine tumor (n=13). **(F)** SET isoform 2 transcripts were overexpressed in normal-tumor tissue pairs but SET isoform 1 remained unchanged. NP, normal pancreas (n=5). PC, pancreatic cancer (n=5). Bars, SD or SEM. *n*= 2 or 3. *p<0.05, **p<0.01, ***p<0.005.

SET is one of the two endogenous inhibitors of the tumor suppressor protein phosphatase 2A (PP2A) (the other being Cancerous Inhibitor of Protein Phosphatase 2A or CIP2A) [3, 4] whose role in EMT is unknown. SET was initially discovered as template-activating factor-I (TAF-I) that regulates histone dynamics as a chaperone, but later studies have identified SET as an inhibitor of histone H4 acetylation [3–5]. Although there are four protein isoforms of SET (Accessions: (1) NP_001116293, (2) NP_003002, (3) NP_001234929, and (4) NP_001234930), each differing only at the N-terminus, isoforms 1 and 2 are the most well-known and correspond to I2βPP2A or TAF-1α and I2αPP2A or TAF-Iβ, respectively (Figure 1A). While the numerous roles of SET are still being explored, recent studies have begun to focus on its protumorigenic roles [3, 4]. Among the key roles include its relationships with Ras-Related C3 Botulinum Toxin Substrate 1 (Rac1) (i.e., Rac1 activation recruits SET to the plasma membrane leading to increased cell migration [6, 7]), the MEK/ERK pathway (i.e., SET regulates cellular proliferation via this pathway [8]), and the c-Jun N-terminal Kinase-1 (JNK) pathway (i.e., SET induces c-Jun/AP-1 activity through changes in c-Jun phosphorylation [9]). In addition to PP2A, SET also inhibits Nm23-H1, a nucleoside diphosphate kinase implicated in granzyme-activated hydrolytic cleavage of DNA [10].

Overexpression of the SET oncoprotein has been reported in numerous cancers, including leukemias, lymphomas, nephroblastoma, hepatoma, and choriocarcinoma, with poor patient outcome correlating with the protein’s expression [4, 11–16]. However, its role in pancreatic cancer remains less understood. Using shRNA library screening, we have earlier demonstrated that knockdown of the SET to suppress growth, reduce colony formation and induce differentiation-promoting microRNAs in pancreatic cancer cells [17]. Subsequently, Farrell et al. showed SET overexpression in human pancreatic cancer to decrease PP2A activity and stabilize c-Myc [18]. In this study, we investigated the putative roles of SET in EMT and pancreatic tumor progression. We report a new role for SET in EMT and revealed the putative molecular pathways involved in SET mediated EMT acquisition in pancreatic cancer.

## RESULTS

### SET isoform 2 is frequently overexpressed in pancreatic cancer

To understand the role of SET in EMT, we identified total SET expression in a panel of pancreatic cancer cell lines. An antibody (SC E-15) which binds to an internal region of SET was used to investigate all SET isoforms (Figure 1A). The results showed total SET protein increased in all pancreatic cancer cell lines tested (except AsPC-1) compared with normal HPDE (Figure 1B-1C). In particular, highest increases were seen in the highly metastatic L3.6pl cell line whereas slower growing AsPC-1 showed least SET expression. To investigate the precise isoform involvement, we further directed our studies on individual isoforms. For differentiating isoforms 1 and 2, specific antibodies were used: one with an epitope for amino acids 1-11 of isoform-1 (ab 97596) and other with an epitope for amino acids 3-18 (ab 1183) of isoform 2. Western Blotting data identify that SET isoform 2, similar to total SET, is overexpressed in all pancreatic cancer cell lines (except AsPC-1) whereas SET isoform 1 was majorly increased only in L3.6pl and Capan-1. Nonetheless, a dimerized form of SET Isoform 1 was seen increased in PANC-1 and MIA PaCa-2 in addition to L3.6pl and Capan-1 (Figure 1B & Supplementary Figure 1A). These results were reproducible with another cell lysis buffer conditions as shown in Supplementary Figure 1B. Next, we used a TaqMan probe for a 100 nt region in exon 8 to identify total SET transcripts while one custom synthesized to probe a 22 nt region (nts 28-49 of the coding sequence) in exon 1 specific to isoform 1 and another pre-synthesized to probe for a 125 nt region (nts 68-193 of the coding sequence) in exons 1-2 specific to isoform 2. Similar to Western blotting results, Real-time PCR analyses showed SET isoform-2 overexpressed in the majority of pancreatic cancer cell lines tested (Figure 1D). The protein levels of isoforms 3 and 4 were not able to be identified since commercial antibodies are not available; on the other hand, qRT-PCR results demonstrated them to be minor isoforms with no significant change between normal and cancerous cell lines (*data not shown*). These data identify that SET isoform 2 is overexpressed in a larger subset of pancreatic cancer cell lines (Figure 1B-1D), whereas SET isoform 1 is overexpressed in a smaller subset of pancreatic cancer cell lines.

To determine the clinical significance of SET overexpression, we examined 23 pancreatic tumor tissues including 18 unmatched pancreatic tumor tissues (compared with 4 normal pancreatic tissues) as mentioned in Supplementary Table 1 (Figure 1E) and 5 matched normal and tumor pancreatic tissues derived from five different human patients (Figure 1F). Consistent with pancreatic cancer cell lines, total SET and SET isoform 2 were significantly overexpressed in PDAC samples and not in other pancreatic tumor subtypes compared with normal pancreatic tissues (Figure 1E). Also, SET Isoform 1 transcripts were also found to be increased in these tissues (Figure 1E). Intriguingly, qRT-PCR analysis of matched normal-tumor tissue pairs showed SET isoform 2 was significantly increased in the entire (5 out of 5) matched tumor samples while no significant differences were observed with SET isoform 1 (Figure 1F). Together these results further supported that SET Isoform 2 is more frequently overexpressed in pancreatic cancer.

### SET Isoform 2 is in the cytoplasm and cell surface while SET isoform 1 is predominantly localized in the nucleus of pancreatic cancer cells

**Figure 2:**
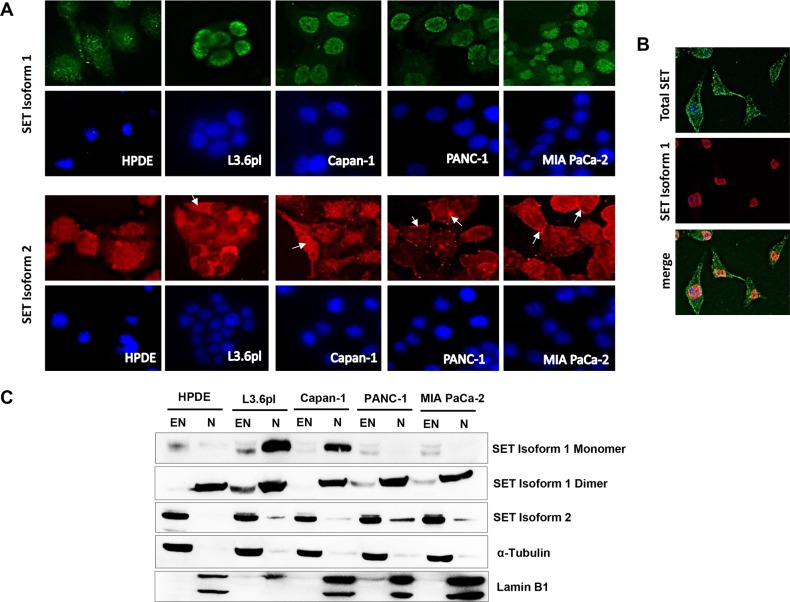
SET isoform 1 is nuclear while SET isoform 2 is extra-nuclear in pancreatic cancer cells **(A and B)** Distinct differences in the cellular distribution of the two SET isoforms in pancreatic cancer cell lines. Immuno-cytochemical analysis confirmed both nuclear and cytoplasmic localization of SET isoform 1 (green) and SET isoform 2 (red) in HPDE whereas nuclear localization of SET isoform 1 and cytoplasmic (L3.6pl, Capan-1) and/or cell surface localization (PANC-1, MIA PaCa-2) of SET isoform 2 (arrows) in pancreatic cancer cell lines. Nuclei are stained with DAPI (blue). Original magnification x60. **(B)** Immuno-cytochemical analysis with Total SET antibody and SET Isoform 1 antibody showed distinct nuclear localization of SET Isoform 1 (red) as opposed to cell surface, cytoplasmic and nuclear localization for total SET (green) in MIA PaCa-2. Nuclei stained with DAPI (blue). **(C)** SET isoform 1 is predominantly confined to the nuclear fractions while SET isoform 2 is predominantly in the extra-nuclear fractions. Cellular fractions were separated from whole cell lysates of pancreatic cancer cells and subjected to Western blotting analysis. Lamin B1 and α-Tubulin were used as markers for nuclear (N) and extra-nuclear (EN) fractions, respectively.

Conducting immunocytochemical analysis using the pan SET E-15 antibody, the distribution of SET isoforms was found to be variable and cell type-dependent. The immunoreactivity appeared nuclear, cytoplasmic, and as well as at the cell surface in pancreatic cancer cell lines (*data not shown*). To precisely investigate differences in isoforms, we used the SET isoform 1 (ab 97596) and isoform 2 (ab 1183) antibodies for immunocytochemical analysis. SET isoform 1 appeared predominantly in the nucleus in all of the pancreatic cancer cell lines examined namely L3.6pl, Capan-1, PANC-1, and MIA PaCa-2 while only a less-intense cytoplasmic staining was noticed (Figure 2A; top panels). In contrast, SET isoform 2 was mainly cytoplasmic and/or at the cell surface in pancreatic cancer cells (Figure 2A; bottom panels).

Further double co-immunolocalization analysis in MIA PaCa-2 cells using a pan-SET antibody (SC E-15) and SET isoform-1 specific antibody (ab 97596) confirmed differential localization characteristics of SET isoforms (Figure 2B). To corroborate these findings biochemically, we also subjected whole cell lysates prepared from various cell lines to subcellular fractionation (nuclear and extra-nuclear) and analyzed by Western blotting. Consistent with the immunocytochemical analysis, Western blotting studies confirmed the nuclear localization of SET isoform 1 in pancreatic cancer cells (Figure 2C). Further, predominant extra-nuclear localization of SET isoform 2 (Figure 2C) was also observed in all the cell lines examined. Overall, these results identified that SET isoform 1 is mainly localized in the nucleus while SET Isoform 2 is localized in the cytoplasm and at the cell surface of pancreatic cancer cells.

### SET Isoform 2 induces EMT and promotes growth, migration, and invasion of pancreatic cancer cells

**Figure 3:**
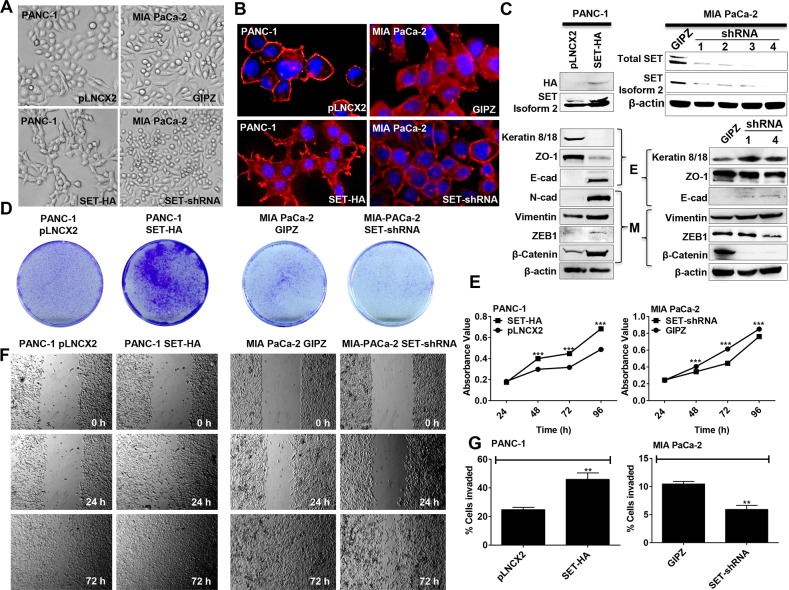
SET isoform 2 induces EMT and promotes growth, migration, and invasion of pancreatic cancer cells **(A-C)** SET isoform 2 (SET-HA) overexpression induces EMT while knockdown of SET (SET-shRNA) suppresses EMT. **(A and B)** Cells with overexpression of SET have a more mesenchymal morphology as compared with control cells (pLNCX2) (A-left panel), and cells with SET knockdown have a more cuboidal, epithelial morphology as compared with control cells (GIPZ) (A-right panel). SET isoform 2 expression also correlated with increased actin stress fibers (red) (B). DAPI is shown (blue). **(C)** Overexpression of SET isoform 2 in PANC-1 and SET knockdown in MIA PaCa 2. Overexpression of SET isoform 2 in PANC-1 increases mesenchymal markers (M) and decreases epithelial markers (E) (except E-cadherin) while knockdown of SET in MIA PaCa-2 partially induces reverse effects. Whole cell lysates (50 μg) were subjected to Western blotting analysis. β-actin, the internal loading control, is shown with a representative blot. **(D and E)** Expression of SET isoform 2 promotes colony formation (D) and cellular proliferation (E) while knockdown of SET in MIA PaCa-2 exerts opposite effects. Cells were grown for 96 h. Growth was measured using a colorimetric assay. **(F)** SET isoform 2 expression promotes migration. Scratched wounds were allowed to heal for up to 72 h. **(G)** Expression of SET isoform 2 promotes invasion. Cells were allowed to invade through the pores of a Matrigel^TM^-coated transwell insert for 48 h. Bars, SEM. *n*=3. *p<0.05, **p<0.01, ***p<0.005.

To further understand the functional relevance of overexpression of SET isoform 2 in pancreatic cancer, we retrovirally overexpressed SET isoform 2 in the inherently epithelial-looking PANC-1 cells and investigated putative changes in cellular morphology and other phenotypes. Retroviral transduction of SET isoform 2 induced a mesenchymal morphology in PANC-1 as judged from elongated cell structures, decreased cell contact, and/or cell spreading within the colonies (Figure 3A-3B). Conversely, knockdown of endogenous SET expression using an shRNA construct against total SET (Figure 3C-top right and Supplementary Figure 2) in the inherently mesenchymal-looking MIA PaCa-2 cell line induced a more epithelial phenotype in MIA PaCa-2 (Figure 3A-3B). SET-induced EMT characteristics in PANC-1 were associated with the disruption of cortical actin bundles with formation of actin stress fibers (Figure 3B) as well as with decreased expression of epithelial markers (Keratin 8/18 and ZO-1) and increased expression of mesenchymal markers (N-cadherin, Vimentin, ZEB1, and β-catenin) (Figure 3C). These characteristics were partially reverted with SET knockdown in MIA PaCa-2 SET-shRNAs compared with control MIA PaCa-2 (GIPZ) with increased formation of actin cortical bundles (Figure 3B) as well as increased expression of epithelial (Keratin 8/18 and E-cadherin) and decreased expression of mesenchymal markers (ZEB1, and β-catenin). The effect on N-cadherin could not be tested since MIA PaCa-2 is an N-cadherin deficient cell line [19, 20] while vimentin and ZO-1 remained unchanged. Nonetheless, the effects of SET on other EMT markers like Keratin 8/18 (epithelial), N-cadherin (in PANC-1) and β-catenin (mesenchymal) were very prominent in both SET overexpression and knockdown cells (Figure 3C). With further investigation, we found increased SET expression to promote colony formation, cellular growth, migration, and invasion, in PANC-1 (Figure 3D-3G), and knockdown of SET to resist these phenotypes in MIA PaCa-2 (Figure 3D-3G; Supplementary Figure 3).

To investigate the possible differential effects of different SET Isoforms on EMT and growth in pancreatic cancer, we transfected SET Isoform 1 overexpressing plasmid tagged with FLAG (SET-FLAG) in PANC-1 (Supplementary Figure 4A) and investigated phenotypic growth and morphological changes as before. Similar to SET Isoform 2, overexpression of SET-FLAG induced mesenchymal morphological changes as compared with control (pCMV3) in PANC-1 judged by elongated morphology of cells and decreased cell contact, albeit at a much lower magnitude than SET isoform-2 (Supplementary Figure 4B). While protein levels of Keratin 8/18 (epithelial marker) was reduced and N-cadherin levels (mesenchymal marker) was increased (Supplementary Figure 4C), no changes were observed with other EMT markers and growth characteristics (Supplementary Figure 4C-4E). Since SET Isoform 2 was more consistently overexpressed in pancreatic cancers (Figure 1) and SET Isoform-2 induced more prominent EMT-related phenotypic changes in PANC-1 as compared with SET Isoform 1 (Figure 3 & Supplementary Figure 4), we focused only on SET Isoform 2 for further mechanistic studies.

### SET-induced EMT is likely N-cadherin driven, involves the Rac1/JNK/c-Jun pathway, and is PP2A-dependent

**Figure 4:**
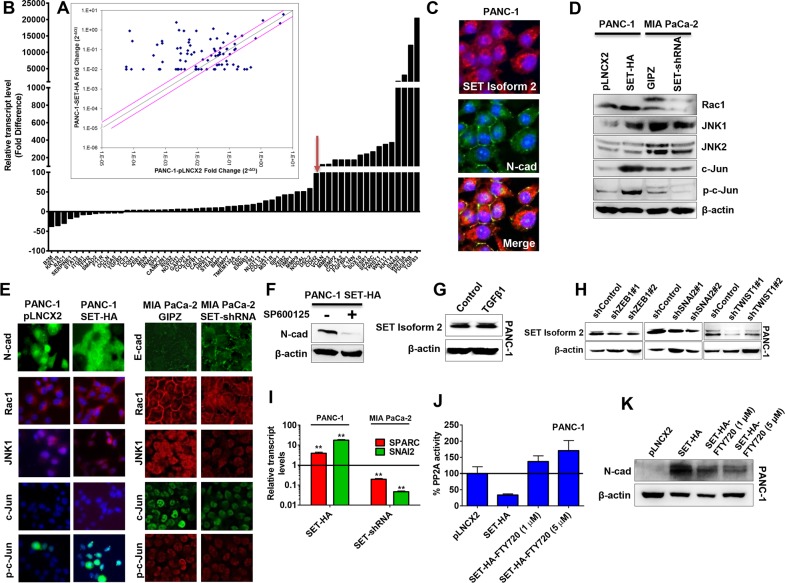
SET induced N-cadherin via the Rac1/JNK/c-Jun signaling pathway **(A and B)** In an RT-PCR profiler array for 84 EMT-related genes, 47 genes were upregulated and 12 genes were down-regulated (with fold changes ≥ 3) in SET overexpression cells (SET-HA) compared with control cells (pLNCX2). A representative scatter plot (A) and genes with respective fold-changes are shown (B). Red arrow indicates CDH2 (N-cadherin gene) as one of the highly upregulated gene (∼100 folds) in SET overexpression cells. **(C)** SET isoform 2 expression (red) partially co-localized with N-cadherin expression (green) at the cell surface (yellow) in PANC-1. **(D)** SET isoform 2 expression alters the protein levels of members in the Rac1/JNK/c-Jun pathway. Oppositely, a reduction in SET led to decreases in Rac1, JNK1, phospho-JNK, c-Jun, and phospho-c-Jun levels. Whole cell lysates (50 μg) were subjected to Western blotting analysis. β-actin, the internal loading control, is shown with a representative blot. **(E)** SET isoform 2 expression alters the cellular organizations and expressions of members in the Rac1/JNK/c-Jun pathway. **(F)** Treating cells with SP600125, a JNK inhibitor, decreased N-cadherin in the PANC-1 SET-HA. **(G)** TGFβ1 (10 ng/mL; 48 h) treatment did not alter SET expression levels in PANC-1. Whole cell lysates (50 - 70 μg) were subjected to Western blotting analysis. β-actin, a loading control is shown with a representative blot. **(H)** Stable knockdown of EMT-regulating transcription factors (ZEB1, SNAI2, and TWIST1) decreases SET protein levels in PANC-1. **(I)** Overexpression of SET in PANC-1 (SET-HA) led to significant increases in both SPARC and SNAI2 transcript levels while opposite effects were observed with the knockdown of SET in MIA PaCa-2 (SET-shRNA) as confirmed with qRT-PCR assays with respective TaqMan probes and GUSB as an internal control. **(J and K)** SET-mediated effects on N-cadherin are PP2A dependent in pancreatic cancer. **(J)** PP2A activity was decreased with SET overexpression (SET-HA) as compared to control (pLNCX2) in PANC-1 while FTY720 (PP2A activator) treatment (1 μM – 5 μM; 24 h – 48 h) in SET-HA rescued SET-mediated decreases in PP2A activity as determined with the PP2A Immunoprecipitation Phosphatase Assay. **(K)**. FTY720 treatment decreased SET-mediated effects on N-cadherin in PANC-1 SET-HA. Whole cell lysates (50 - 70 μg) were subjected to Western blotting analysis. β-actin as a loading control is shown with a representative blot. Bars, SD or SEM. n = 2-3.

To better understand SET regulation of EMT, we performed a PCR array for 84 genes known to be involved in EMT. These included, but were not limited to, genes involved in cell adhesion, migration, cellular differentiation, morphogenesis, development, growth, and proliferation (Supplementary Table 2). With the overexpression of SET Isoform 2 in PANC-1, we found at least 47 genes to be upregulated and 12 genes to be downregulated with a fold difference of at least 3, as compared with control cells (pLNCX2 PANC-1) (Figure 4A-4B, Supplementary Table 3). Interestingly, we identified significant increases in N-cadherin transcript levels by ∼ 100 folds as indicated in Figure 4B. These results were consistent to our earlier results where we observed SET overexpression (Figure 3C) to significantly increase N-cadherin protein levels in PANC-1. E-cadherin transcript and protein levels were also increased in PANC-1 overexpressing SET (Figures 4B & 3C), albeit at a lower magnitude as compared with N-cadherin. Since gain of N-cadherin is a characteristic feature of EMT and previous studies suggest expression of non-epithelial cadherins can downregulate the cell surface expression of E-cadherin [21, 22], we speculated that SET induced expression of N-cadherin mainly influenced EMT in PANC-1. Further, immunocytochemical analysis of N-cadherin and SET expressions in PANC-1 identified partial co-localization of N-cadherin and SET-isoform-2 at the cell surface (Figure 4C), although co-immunoprecipitation assays showed no direct interaction between the two proteins (*data not shown*).

Since N-cadherin has been shown to act downstream of Rac1 and JNK1 [23, 24], we hypothesized that SET would act through the Rac1/JNK1 pathway to induce N-cadherin expression. Furthermore, SET was found to increase Rac-1 expression and during cellular migration has been shown to shuttle to the cell surface to interact with Rac-1. To test this hypothesis, we analyzed the expression of members of the Rac1/JNK1pathway in cells overexpressing SET (PANC-1 SET-HA) compared with control. Clearly, along with an increase in SET, Rac1, JNK1, c-Jun, and phospho-c-Jun were also increased in PANC-1 SET-HA (Figure 4D-4E). However, in cells where SET was knocked down (MIA PaCa-2 SET-shRNA), Rac1 expression was only slightly decreased; but significant reorganization within cells was noticed by immunocytochemical analysis (Figure 4E). JNK1, c-Jun, and phospho-c-Jun expressions were also reduced in MIA PaCa-2 SET-shRNA (Figure 4D-4E). These results suggested that SET induced N-cadherin expression is likely mediated through the Rac1/JNK1/c-Jun pathway. To further probe whether JNK1 activation and phosphorylation of c-Jun is a required event for SET induction of N-cadherin expression, we treated PANC-1 SET-HA cells with a selective ATP-competitive inhibitor of JNK (SP600125) and assayed for N-cadherin levels. As shown in Figure 4F, SP600125 decreased N-cadherin in PANC-1-SET-HA, suggesting an essential role for JNK and phospho-c-Jun in SET activation of N-cadherin.

In addition to N-cadherin, PCR array results showed significant increase in the expressions of TGFβ ligands (TGFβ1, TGFβ3), SPARC (Secreted Protein Acidic and Rich in Cysteine) and several EMT-promoting transcription factors (SNAI1, SNAI3, TWIST1, ZEB1, and ZEB2) in SET overexpressing PANC-1 cells. Thus we next examined whether SET proteins can act as putative mediators of TGFβ signaling pathway. We treated PANC-1 (a TGFβ1-responsive cell line) with a recombinant human TGFβ1 (48 h; 10 ng/mL) and interrogated for possible changes in SET protein levels. However, SET protein levels were found to be unaltered in TGFβ-treated cells (Figure 4G) suggesting SET may not be involved in the TGFβ-driven EMT process. However, when interrogated for alterations in SET protein levels in PANC-1 clones knocked down for various EMT-driving transcription factors (ZEB1, SNAI1, SNAI2, and TWIST1), we found significant decreases in SET isoform-2 protein levels in ZEB1, SNAI2 & TWIST1 knockdown PANC-1 cells (Supplementary Figure 5; Figure 4H) suggesting that SET is integrally related to the transcription factor machineries governing the EMT process.

Conversely, when tested for putative SET target genes in SET knockdown MIA PaCa-2 cells, significant reductions were noticed with SPARC and SNAI2 (Slug) (Figure 4I) that causes transcriptional repression of E-cadherin. Increased expression of E-cadherin at the cell surface in contrast with the decreased expression of Slug was also seen by immunocytochemical analysis of MIA PaCa-2-SET-shRNA cells (Supplementary Figure 6). We were not able to detect SPARC (osteonectin) at the protein levels perhaps due to low expression levels. These results suggested that E-cadherin expression in MIA PaCa-2-SET-shRNA is most likely mediated through transcriptional repression of SPARC and SNAI2 (Slug). Further, given the lack of N-cadherin expression in MIA PaCa-2 [19, 20], these results suggested that increased E-cadherin expression as a dominant pathway by which SET knockdown inhibited EMT in these cells.

It is also well-known that SET is one of the endogenous inhibitors of the tumor suppressor PP2A and PP2A itself has been shown to govern the epithelial characteristics of solid tumor cell lines [25, 26]. Hence to experimentally verify whether SET-mediated effects on EMT and N-cadherin are PP2A-dependent, we tested PP2A activity in SET overexpressing PANC-1 cells. As expected, we found PP2A activity to be decreased (∼70%) in SET overexpressing cells (Figure 4J). To check whether SET-mediated effects are PP2A-dependent, we treated SET overexpressing PANC-1 cells with FTY720 (Fingolimod), an activator of PP2A that interferes with SET-PP2A interaction [27]. Indeed, Fingolimod treatment not only enhanced PP2A activity (Figure 4J) but also partially reversed SET-mediated increase in N-cadherin levels (Figure 4K). Taken together, these results defined that the SET effects on EMT and N-cadherin are probably mediated by multiple players including the Rac1/JNK1/c-Jun pathway, PP2A activity and key EMT-driving transcriptional factors.

### SET Isoform 2 induces tumor growth and metastasis in an orthotopic xenograft pancreatic tumor model

**Figure 5:**
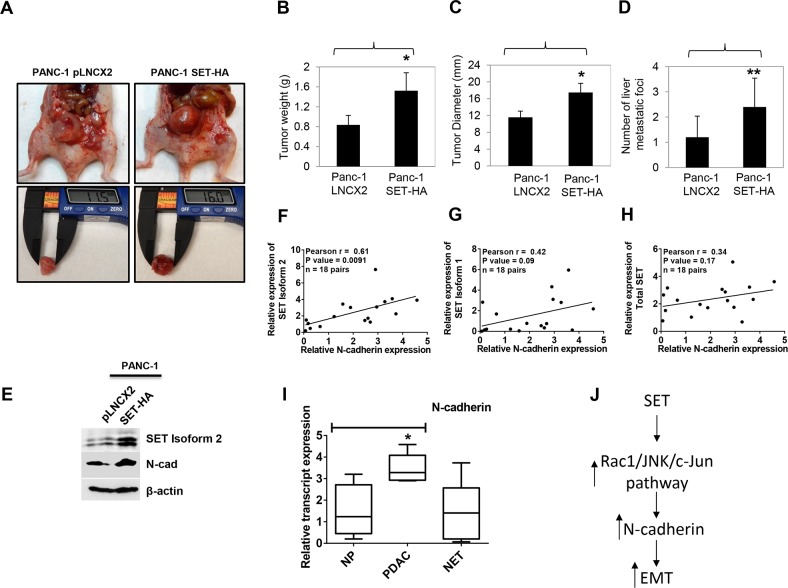
SET isoform 2 overexpression increased tumor growth and metastasis in orthotopic pancreatic cancer mice model **(A-D)** Mice (n=3) injected with PANC-1 SET-HA cells developed larger (A & C) and heavier (B) tumors than PANC-1 pLNCX2 mice. D. SET isoform 2 overexpression led to increased number of metastatic foci in mouse livers. **(E)** SET isoform 2 expressing tumors showed increased N-cadherin protein expression. **(F-H)** N-cadherin positively correlated with SET in the order SET Isoform 2 (F) > SET Isoform 1 (G) > Total SET (H) in 18 pairs of pancreatic cancer tissues. **(I)** N-cadherin transcripts (CDH2) are overexpressed in PDAC but not in NET tissue samples similar to SET Isoform 2 transcripts as in Figure 1E. NP, normal pancreas (n=4). PDAC, pancreatic ductal adenocarcinoma (n=5). NET, neuroendocrine tumor (n=13). **(J)** Schematic representation of SET induced N-cadherin-driven EMT in pancreatic cancer. Bars, SD or SEM. *p<0.05, **p<0.01.

To assess *in vivo* relevance, PANC-1 SET HA cells were injected into the pancreas of athymic nude mice, and tumor burden and metastatic spread were analyzed by monitoring the mice for 10 weeks. Mice with orthotopic tumor implantation of PANC-1 SET-HA gained approximately 2-4 g over 10 weeks post-surgery. As seen in Figure 5A-5C, primary tumor weight and diameter was found to increase with SET expression with an average increase of 1.93±0.85-fold (p<0.05) and 1.51±0.06-fold (p<0.05), respectively, in SET overexpressing tumors. Furthermore, the number of metastatic foci significantly increased on the liver surface with SET overexpression by 2.4±1.14-fold (p<0.01) compared with control vector expressing tumors (Figure 5D). Western blotting analysis of tumor lysates prepared from SET overexpressing tumors showed increased expression of N-cadherin at the tumor edges suggesting its primary involvement in the formation of secondary tumors (Figure 5E). Since the effect of SET overexpression was pronounced in increasing N-cadherin levels, we carried out correlation studies for SET and N-cadherin expression levels. First, we checked the expression levels of N-cadherin in the same panel of 18 unmatched human pancreatic tumor tissues (that was earlier utilized in Figure 1E) to determine if they correlated with SET expression. Consistently, we determined a significant positive correlation between SET Isoform 2 and N-cadherin with a Pearson r value of 0.61 (p value = 0.0091) (Figure 5F). In addition, SET Isoform 1 (r = 0.42, p = 0.09) (Figure 5G) as well as total SET (r = 0.34, p = 0.17) (Figure 5H) transcripts positively correlated with N-cadherin, although to a lesser extent as compared with SET Isoform 2 and was not statistically significant. Moreover, similar with the SET overexpression, we found N-cadherin levels to be significantly increased in PDAC samples (Figure 5I). No significant correlation was obtained with SET and E-cadherin in pancreatic cancer tissues (r = 0.14, p = 0.56) (Supplementary Figure 7). Overall, these data support that SET isoform 2 overexpression in pancreatic cancer promotes N-cadherin expression, tumor growth, and metastasis *in vivo*.

## DISCUSSION

We earlier reported SET knockdown to reduce growth and colony formation of pancreatic cancer cells [17]. Interestingly, we also found SET knockdown to increase the epithelial features of pancreatic cancer cells by promoting compact colony morphology with smooth colony edges and increased cell-to-cell contacts. Since these observations suggested that endogenous inhibition of PP2A could play an integral role in tumor progression, it prompted us to further investigate the role of SET in EMT in pancreatic cancer. Moreover, a recent study by Wang et al. also reported expression of CIP2A (another endogenous inhibitor of PP2A) to correlate with altered expression of EMT markers in pancreatic cancer, although a causal relationship between CIP2A and EMT acquisition was not investigated [28]. In this study, we demonstrate SET isoform 2 as the predominantly overexpressed form of SET in pancreatic cancer and that it triggers N-cadherin expression for acquisition of EMT characteristics possibly through the Rac-1/JNK/c-jun pathway (Figure 5J). In addition, SET inhibited PP2A activity and induced several key EMT-driving transcriptional factors to facilitate EMT of pancreatic cancer cells. Stable overexpression of SET isoform 2 promoted mesenchymal characteristics in epithelial-looking cells while knockdown of SET mitigated such features in mesenchymal-looking cells. *In vivo*, SET isoform 2 overexpression augmented tumor growth and metastasis in an orthotopic mouse model. Taken together, these findings illustrate a novel role for SET in EMT and suggest that SET oncoprotein may be utilized as a therapeutic target to minimize EMT and metastasis in a subset of pancreatic cancer patients.

Although there are four known isoforms of human SET, their individual roles in tumor development and progression remain obscure. Each of the isoforms only differs at the N-terminus with no variations in the PP2A inhibitory, chromatin remodeling, and acidic C-terminal domains. Also, a putative nuclear localization signal (NLS) sequence is commonly identified in all 4 isoforms. However, distinct differences in the nuclear and extra-nuclear localizations of SET isoform 1 and SET isoform 2 in normal and cancerous pancreatic cancer cells were noted in this study. Further, SET isoform 2 itself exhibited cell type-dependent, cytoplasmic and cell surface localization observed in pancreatic cancer cells. Based on these observations, it seems likely that either the variable N-terminal region or putative modification(s) of the N-terminal region (in the case of SET isoform 2) decides the nuclear/extra-nuclear localization of SET in normal and cancer cells.

Indeed, early *in vivo* phosphorylation and radiosequencing studies have identified two putative serine residues (Ser-9 and Ser-24) within the N-terminus of SET isoform 2 to undergo phosphorylation in a protein kinase C (PKC) dependent manner. Serine phosphorylation of SET prevents dimerization of the protein resulting in the extra-nuclear escape to regulate Rac1 binding in HeLa and HEK293 cells. Our findings are consistent in that the phosphorylation of the N-terminal serine residues are increased in a subset of pancreatic cancers, allowing for increased extra-nuclear translocation of SET isoform 2 and accumulation at the cell surface. More importantly, our findings identify that the differential localization of SET isoform 2 influences the indolent epithelial characteristics and aggressive mesenchymal characteristics of tumor cells. In contrast to SET Isoform 2, SET Isoform 1 was observed to be predominantly dimerized and located within the nucleus and thereby probably isoform 1 is likely to have less prominent role in EMT regulation in cancer cells as compared with isoform 2. Supportive evidence came from examining the pancreatic cancer cell lines (PANC-1 and MIA PaCa-2) with abundant expression of SET isoform 2 which correlated with high Rac1 expression at the cell surface. Consistently, these cells are more migratory and invasive when compared with their counterparts (e.g., AsPC-1) which exhibited lower abundance of SET and Rac1 expression. It was also interesting to note that SET overexpression alone activated mesenchymal characteristics in three epithelial-looking cell lines (PANC-1, L3.6pl, and Capan-1) even though these lines already exhibited high endogenous SET expression. While Rac1-driven mechanisms have been shown to recruit SET to the cell surface to promote cellular migration, the current study reveals SET activation without Rac1 perturbation was sufficient to increase migratory capabilities. Whether SET increases Rac1 activity to increase migratory capabilities in pancreatic cancer remains unknown; however, it is likely that both SET overexpression and Rac1-induced SET recruitment to the cell surface can concomitantly amplify migratory signals during EMT.

In order to inhibit SET isoform 2, we utilized an shRNA construct that we identified in an shRNA library screen to increase epithelial characteristics of pancreatic cancer cells [17]. However, this shRNA targeted all 4 human SET isoforms and induced a dramatic inhibition of total SET with extensive reorganization of Rac1 in mesenchymal cells. The SET-inhibited cells appeared highly epithelial and organized into well-defined spheroids in soft-agar cultures which was distinctly different from the scrambled shRNA-expressing MIA PaCa-2 cells that appeared more plastic with both epithelial and mesenchymal characteristics. In addition, overexpression of SET Isoform 1 alone also induced EMT-like characteristics though to a lesser extent as compared with Isoform 2. These results also supported that the combined inhibition of SET isoforms could have even more drastic effects in resisting EMT than inhibiting SET isoform 2 alone. Very recently, the JNK pathway has been shown to be a key player in the progression of EMT-associated cellular phenotypic changes that required transcriptional reprogramming of critical EMT genes [29]. Our studies revealed SET isoform 2 induction of N-cadherin to operate through the JNK1 pathway (phosphorylating c-Jun), and both shRNA knockdown of SET and pharmacological inhibition of JNK pathway to diminish N-cadherin activation and EMT characteristics. Other groups have identified agents such as transglutaminase 2 and collagen to upregulate N-cadherin via JNK1 activation to promote EMT in pancreatic cancer cells [23, 24, 30, 31]. Thus, our data lends further credence to these findings and provide a new molecular link between SET, JNK, and N-cadherin for activation of EMT in pancreatic cancer cells.

Although SET effects on N-cadherin expression was most prominently observed in this study, it is also likely that other additional mechanisms could contribute to SET effects on pancreatic cancer cells. In this regard, we found SET inhibition of endogenous PP2A activity as partially responsible for the increased N-cadherin expression. We also found SET to induce the expression of several key transcriptional factor genes viz., ZEB1, SNAI2, and TWIST1 that are well known to drive EMT characteristics in solid tumors. Conversely, shRNA knockdown of these EMT-driving transcription factors reduced SET levels thereby identifying a possible interplay of SET in ZEB1/SNAI2/TWIST1-induced EMT. While these results highlight the multifarious roles of SET in coordinating the EMT process, understanding the precise role of EMT-driving transcription factors in regulating SET, or vice versa, warrants further experimentation. Further, while silencing SET in MIA PaCa-2 clearly increased E-cadherin protein expression as well as epithelial characteristics, paradoxically, overexpression of SET in PANC-1 did not decrease E-cadherin expression. Instead, SET expression showed an increase in E-cadherin protein in PANC-1 albeit to a lesser extent than that observed with N-cadherin. Further, no significant correlations of SET and E-cadherin were obtained in pancreatic cancer cell lines (*data not shown*) and tumor tissues (Supplementary Figure 7). At present, the reasons for these discrepancies in E-cadherin expression and EMT characteristics are unclear. However, it has been shown earlier that increased expression of non-epithelial cadherins (e.g., N-cadherin) could downregulate the cell surface expression of epithelial cadherins (e.g., E-cadherin) and hence, it is possible that increased N-cadherin expression in PANC-1-SET cells could have overridden the effects of E-cadherin to drive EMT in these cells. In case of MIA PaCa-2 with no N-cadherin expression, any increase in E-cadherin expression could have played a dominant role in inducing epithelial characteristics as a consequence of SET knockdown. Finally, the extent of cellular morphological changes with SET overexpression also varied somewhat with cell types with clear appearance of cell elongation, membrane ruffling, and cytoskeletal remodeling (e.g., PANC-1) to mere appearance of cell spreading with no significant alterations in individual cell morphology (e.g., Capan-1). Also, attempts to significantly knockdown SET isoform 2 (>50% reduction) in some epithelial cell lines (e.g., Capan-1 and L3.6pl) were futile and precluded our understanding of the effect of SET loss in epithelial lines. While very high levels of SET in L3.6pl and Capan-1 could be contributing factors, the precise reasons for such inconsistencies are unclear at present. In addition, the presence of CIP2A and its possible roles in supporting survival and EMT characteristics may be a few confounding factors. In this regard, we note that Capan-1 and L3.6pl also express high levels of CIP2A, and emerging studies are reporting CIP2A to independently promote EMT characteristics in many solid tumors including bladder, cervical and renal [32–34].

In summary, our study has delineated the putative mechanism by which SET increases N-cadherin and its ramifications on the hallmarks of EMT in pancreatic cancer. As increased N-cadherin alone is capable of increasing the migratory and invasive behaviors of pancreatic cancer cells, and SET is identified to activate N-cadherin, it supports a causal relationship for SET in promoting cadherin switching, EMT, and metastasis in pancreatic cancer. SET isoform 2 overexpression also had significant consequences on the primary and secondary pancreatic tumor growth *in vivo*, and hence may be utilized for early prediction of tumor aggressiveness and metastasis. Since targeting SET at the cell surface is pharmacologically tractable [18, 35], further studies in this direction are likely to identify new opportunities for intervening pancreatic tumor progression.

## MATERIALS AND METHODS

### Reagents and antibodies

Fetal bovine serum (FBS), 4′,6′-diamidino-2-phenylindole (DAPI), propidium iodide, ethylene glycol bis(2-aminoethyl ether) tetraacetic acid (EGTA), phenylmethanesulfonyl fluoride (PMSF), N-ethyl maleimide (NEM), sodium orthovanadate (Na_2_VO_4_), and iodoacetamide were obtained from Sigma Aldrich (St. Louis, MO). The bicinchoninic acid (BCA) protein assay reagent and West Pico Chemiluminescent substrate were from Pierce Chemical (Rockford, IL). FTY720 (Fingolimod) was purchased from Cayman chemical (Ann Arbor, MI). Fluorescent anti-fade mounting reagent was obtained from Molecular Probes (Invitrogen, Life Technologies, Carlsbad, CA). Plasticware for cell culture was obtained from Corning (Corning, NY). All cell culture media were purchased from Mediatech (Manassas, VA) except for the human keratinocyte basal medium which was procured from Molecular Probes.

The anti-human goat polyclonal I2PP2A (E-15), rabbit polyclonal N-cad (H-63) for immunocytochemistry, c-Jun (H-79), and JNK2 (N-18), and mouse monoclonal JNK1 (F-3) and p-c-Jun (KM-1) antibodies were obtained from Santa Cruz Biotechnology (Santa Cruz, CA). The anti-human rabbit polyclonal E-cad for Western blotting (ab53033), SET isoform 1 (ab97596), and SET isoform 2 (ab1183) antibodies were from Abcam (Cambridge, MA). The anti-human rabbit polyclonal α-Tubulin (2144S) and rabbit monoclonal vimentin (D21H3; 5741) and Lamin B1 (D9V6H; 13435) antibodies were purchased from Cell Signaling Technology (Danvers, MA). EMT antibody kit (9782) was also purchased from Cell Signaling Technology to detect all EMT markers. The anti-human mouse N-cad for Western blotting (610920) and Rac1 (610651) antibodies were obtained from BD Biosciences (San Jose, CA). The mouse monoclonal anti-β-actin antibody (A1978) and Anti-c-Jun (A5968) was purchased from Sigma-Aldrich. The rabbit polyclonal anti-HA antibody and HRP-conjugated secondary antibodies were from Bethyl Laboratories (Montgomery, TX). The mouse monoclonal antibody for FLAG (635691) was procured from Clontech (CA). The anti-human mouse monoclonal E-cad antibody for immunocytochemistry was kindly provided by Dr. Parmender Mehta of the University of Nebraska Medical Center (Omaha, NE).

### Cell culture

The pancreatic cancer cell lines AsPC-1, Capan-1, HPAF-II, MIA PaCa-2, and PANC-1 were received from ATCC (Manassas, VA). These cell lines were propagated, expanded, and frozen immediately upon receipt. The cells revived from the frozen stock were used within 10-20 passages, not exceeding a period of 2-3 months. The ATCC uses morphological, cytogenetic, and DNA profile analyses for characterization of cell lines. Human pancreatic ductal epithelial (HPDE) cells were kindly received from Dr. Ming Tsao of the Ontario Cancer Institute (Toronto, Canada). The L3.6pl cell line was kindly received from Dr. Isiah D. Fidler at The University of Texas MD Anderson Cancer Center (Houston, TX). Both HPDE and L3.6pl cell lines were handled as other cell lines and were genotyped by DNA fingerprinting (PowerPlex 16; Promega, Madison, WI) as per the manufacturer’s instructions. The growth conditions of all cell lines were performed as described previously [36].

### Tumor RNA and tissues

Total RNA for PDAC and normal pancreatic tissues were obtained from OriGene (Rockville, MD). Matched PDAC and normal adjacent tissues were procured from the National Disease Research Interchange (Philadelphia, PA) [36]. The demographic and clinical information available for these samples are shown in Supplementary Table 1. The NDRI obtains written consents for research use from the sources. The procurement and use of these human tissues was done in accordance with the University of Georgia Institutional Review Board. The Board has determined that the use of human biological tissues in this research does not meet the criteria for research involving human subjects per 45 CFR 46.102, and therefore does not require human subject approval by the Board.

### Quantitative real-time PCR, Western blot analysis, immunocytochemistry, cellular proliferation assay, and colony formation assay

These procedures were performed as described earlier [17, 36, 37]. TaqMan primers and probes for SET (Hs00853870_g1), SET isoform 1 (custom synthesized to nucleotides 28-49 of the CDS), SET isoform 2 (Hs01118851_m1), CDH1 (Hs01013959_m1), CDH2 (Hs00983062_m1), ZEB1 (Hs00232783_m1), SNAI2 (Hs00950344_m1), TWIST1 (Hs01675818_s1), SPARC (Hs00234160_m1), and GusB (Hs99999908_m1) were obtained from Applied Biosystems (Life Technologies). For Western blotting analysis, cells were lysed either in TNE (Tris-NP40-EDTA) lysis buffer (10 mM Tris Base pH 8.0, 0.5% NP-40, 2 mM EDTA, 2 mM PMSF supplemented with a cocktail of protease and phosphatase inhibitors) or in HEPES lysis buffer (20 mM HEPES pH 7.4, 150 mM NaCl, 2 mM EDTA, 10 mM NaF, 10% glycerol, 1% NP-40 supplemented with a cocktail of protease and phosphatase inhibitors) and samples were prepared. To study the cellular distribution of SET isoforms, nuclear and extra-nuclear fractions from cell lysates were prepared as per previously published procedures [38].

### Wound healing assay

30×10^4^ cells were seeded in a 6 cm dish and grown until 90-95% confluency. A single scratch across the dish was then made using a sharp edge. Images of the wound were taken at 0, 24, 48, and 72 h at 8x magnification using a Nikon AZ100 Multizoom microscope.

### EMT PCR array analysis

The human Epithelial to Mesenchymal Transition RT^2^ Profiler^TM^ PCR Array (PAHS-090ZC-2) was purchased from Qiagen (Venlo, Netherlands). The recommended RT^2^ First Strand Kit (330401) and RT^2^ SYBR® Green ROC^TM^ qPCR Master Mix (330520) were used as per the manufacturer’s instructions.

### Transwell assay

2.5×10^4^ cells in serum-free medium were seeded into the upper chamber of a 24-well transwell insert with 8.0 μm PET membrane pores and a BD BioCoat^TM^ Matrigel^TM^ coating (Corning). Bottom wells were filled with complete medium. Cells were allowed to invade through the pores for 48 h under cell culture conditions. After incubation, the invaded cells that adhered to the bottom of the membrane were then fixed with methanol and stained with crystal violet. Cells from the upper surface of the filter were removed by scraping with a cotton swab. The number of cells that penetrated the membrane was determined by counting the mean cell number of five randomly selected high-power fields.

### Plasmid construction and retroviral gene transfer

The GIPZ Lentiviral mouse and human SET shRNA (Thermo Scientific, Waltham, MA) as well as ZEB1, SNAI2, and TWIST1 shRNA constructs (Dharmacon, Lafayette, CO) were obtained and utilized as previously described [17]. Detailed characterization of as ZEB1, SNAI2, and TWIST1 shRNA knockdown clones will be described elsewhere. The pcDNA-SET-HA construct for SET Isoform 2 was obtained from Dr. Judy Lieberman via AddGene (Plasmid 24998). The insert was then subsequently cut out of the pcDNA3.1 vector using BamHI and XhoI and ligated to the pLNCX2 vector at the BglII and SalI sites. The empty vector was used as a control. Transfection into the packaging cell line and subsequent infection of the viral particles into PANC-1 were performed as described earlier [36]. Cells were selected and maintained with G418 (800 and 200 μg/ml, respectively). The pCMV3-SET-FLAG and control vector (HG13009-NF) for SET Isoform 1 was purchased from Sino Biological Inc. (Beijing, China).

### PP2A phosphatase activity assay

PANC-1 cells with SET overexpression (SET-HA) or control (pLNCX2) under indicated conditions were washed with cold PBS (pH 7.4) and then lysed with TNE lysis buffer as mentioned previously. Cell lysates were centrifuged (15,000 rpm; 15 minutes) and supernatants were protein estimated with BCA assay. Subsequently, 300 μg of cell lysates were subjected to PP2A phosphatase activity assay using the PP2A Immunoprecipitation Phosphatase Assay Kit (Millipore) as per manufacturer’s instructions.

### Animal studies

All animal experiments were performed in accordance with the Animal Care and Use Procedures at the University of Georgia, and the experimental protocol was approved by the Institutional Animal Care and Use Committee (IACUC) at the University of Georgia. Eight-week old female NU/J athymic nude mice were purchased from The Jackson Laboratory (Bar Harbor, ME). The mice underwent an established surgical method for an orthotopic injection into the pancreas [39]. In brief, mice were anesthetized with ketamine-xylazine (100 and 20 mg/kg, respectively), and the abdominal skin and muscle layers were incised (approximately 1 cm) slightly medial to the splenic silhouette. The pancreas was gently retracted and positioned to allow for the injection of 1×10^6^ cells in 50 μl of sterile HBSS containing 1% (v/v) Matrigel. After solidification of the Matrigel (approximately 2 min), the pancreas was placed back into the abdominal cavity, and both the muscle and skin layers were closed with interrupted sutures. Following recovery from surgery, mice were monitored for 10 weeks.

As per the protocol, metastatic progression was determined by harvesting the primary tumor and liver. After examining for signs of metastasis, tumor volumes were measured and calculated as the mean of the three dimensions (length x width x depth). The tissues were then snap frozen and processed for biochemical and histological examinations.

### Statistical analysis

GraphPad Prism 6 (GraphPad software Inc.) was used to carry out data analysis. The student’s t test between two experimental groups, or one-way or two-way analysis of variance (ANOVA) with multiple comparisons test was used to determine statistical significance. Unless otherwise indicated, *p*<0.05, *p*<0.01, and *p<0.005* compared with control conditions were represented by one, two, or three asterisks, respectively.

## SUPPLEMENTARY TABLES AND FIGURES




