# Supplementary material for: SET contributes to the epithelial-mesenchymal transition of pancreatic cancer
Source: Oncotarget. 2017 Jul 7;8(40):67966–79. doi: 10.18632/oncotarget.19067 (PMC5620228; doi:10.18632/oncotarget.19067)
Supplement: Supplementary file 1 [file oncotarget-08-67966-s001.pdf]

SET Contributes to the epithelial-mesenchymal transition of pancreatic cancer

SUPPLEMENTARY MATERIALS

Supplementary Table 1: Clinical and histological characterization of human pancreatic tumor samples

Sample	Gender	Age	Appearance	Diagnosis	Tumor grade	Stage
N1	Male	66	Normal	Adenocarcinoma of pancreas	AJCC G2: Moderately differentiated	Normal
N2	Male	37	Normal	Pancreatitis, chronic	NULL	Normal
N3	Male	74	Normal	Tumor of pancreas, intraductal, papillary, mucinous	Other Histologic Grade	Normal
N4	Female	57	Normal	Tumor of pancreas, Islet cell	Not Reported	Normal
C1	Female	79	Tumor	Adenocarcinoma of pancreas, ductal	AJCC G3: Poorly differentiated	IB
C2	Male	57	Tumor	Adenocarcinoma of pancreas	AJCC G2: Moderately differentiated	IIB
C3	Male	75	Tumor	Adenocarcinoma of pancreas, ductal	AJCC G2: Moderately differentiated	IIB
C4	Female	71	Tumor	Adenocarcinoma of pancreas, ductal	AJCC G3: Poorly differentiated	III
C5	Male	69	Tumor	Adenocarcinoma of pancreas, ductal	AJCC G3: Poorly differentiated	III
C6	Female	69	Tumor	Tumor of pancreas, neuroendocrine	AJCC G1: Well differentiated	Not Reported
C7	Female	73	Tumor	Tumor of pancreas, neuroendocrine, metastatic	AJCC G1: Well differentiated	Not Reported
C8	Female	60	Tumor	Tumor of pancreas, neuroendocrine	Not Reported	Not Reported
C9	Male	50	Tumor	Tumor of pancreas, Islet cell	Not Reported	Not Reported
C10	Male	66	Tumor	Tumor of pancreas, neuroendocrine	Not Reported	Not Reported
C11	Female	64	Tumor	Tumor of pancreas, neuroendocrine	Not Reported	Not Reported
C12	Female	60	Tumor	Tumor of pancreas, neuroendocrine	Not Reported	Not Reported
C13	Female	60	Tumor	Tumor of pancreas, neuroendocrine	Not Reported	Not Reported
C14	Female	61	Tumor	Tumor of pancreas, neuroendocrine	Not Reported	Not Reported
C15	Male	51	Tumor	Tumor of pancreas, Islet cell	Not Reported	Not Reported
C16	Male	31	Tumor	Tumor of pancreas, Islet cell	Not Reported	Not Reported
C17	Male	76	Tumor	Tumor of pancreas, Islet cell	Not Reported	Not Reported
C18	Female	57	Tumor	Tumor of pancreas, neuroendocrine	Not Reported	Not Reported

Supplementary Table 2: Genes profiled for RT-PCR profiler array

AHNAK	AKT1	BMP1	BMP2	BMP7	CALD1	CAMK2N1	CAV2	CDH1	CDH2	COL1A2	COL3A1
COL5A2	CTNNB1	DSC2	DSP	EGFR	ERBB3	ESR1	F11R	FGFBP1	FN1	FOXC2	FZD7
GNG11	GSC	GSK3B	IGFBP4	IL1RN	ILK	ITGA5	ITGAV	ITGB1	JAG1	KRT14	KRT19
KRT7	MAP1B	MMP2	MMP3	MMP9	MSN	MST1R	NODAL	NOTCH1	NUDT13	OCLN	PDGFRB
PLEK2	PPPDE2	PTK2	PTP4A1	RAC1	RGS2	SERPINE1	SIP1	SMAD2	SNAI1	SNAI2	SNAI3
SOX10	SPARC	SPP1	STAT3	STEAP1	TCF3	TCF4	TFPI2	TGFB1	TGFB2	TGFB3	TIMP1
TMEFF1	TMEM132A	TSPAN13	TWIST1	VCAN	VIM	VPS13A	WNT11	WNT5A	WNT5B	ZEB1	ZEB2
ACTB	B2M	GAPDH	HPRT1	RPLP0	HGDC	RTC	RTC	RTC	PPC	PPC	PPC

Supplementary Table 3: List of EMT-related genes (including house-keeping genes) from a RT-PCR profiler array with fold changes on SET overexpression (SET-HA) compared with control cells (pLNCX2) in PANC-1.

See Supplementary File 1

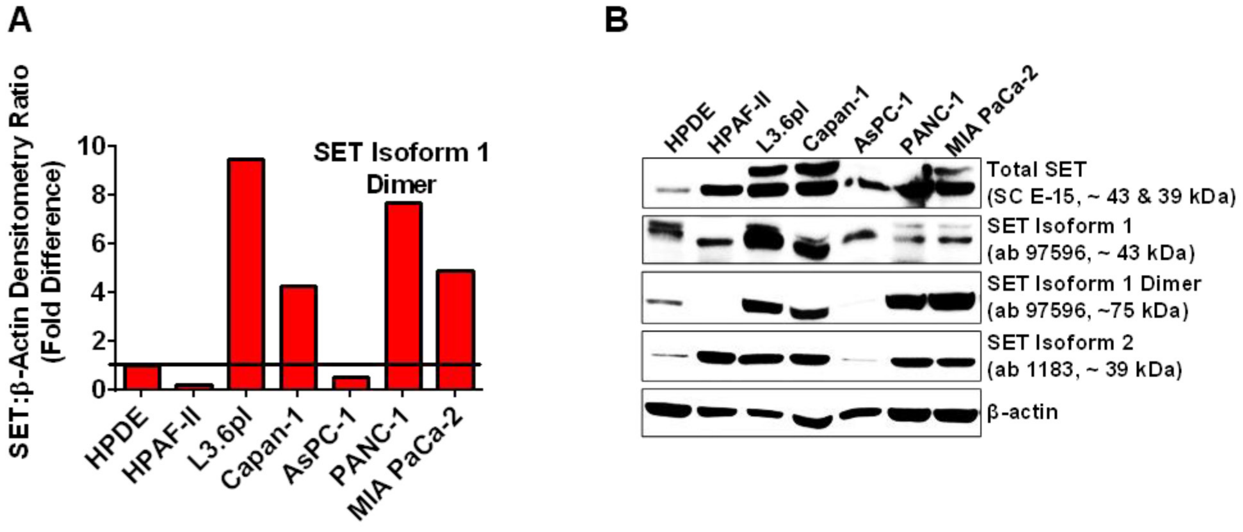

Supplementary Figure 1: (A) The bands of SET Isoform 1 dimer from Fig. 1B were quantified by densitometry and SET:β-actin ratios plotted. **(B)** Protein levels of Total SET, SET isoform 1, and SET isoform 2 in a panel of pancreatic cancer cells. Whole cell lysates (50 µg) prepared with HEPES Lysis Buffer were subjected to Western blotting analysis. β-actin, the internal loading control, is shown with a representative blot.

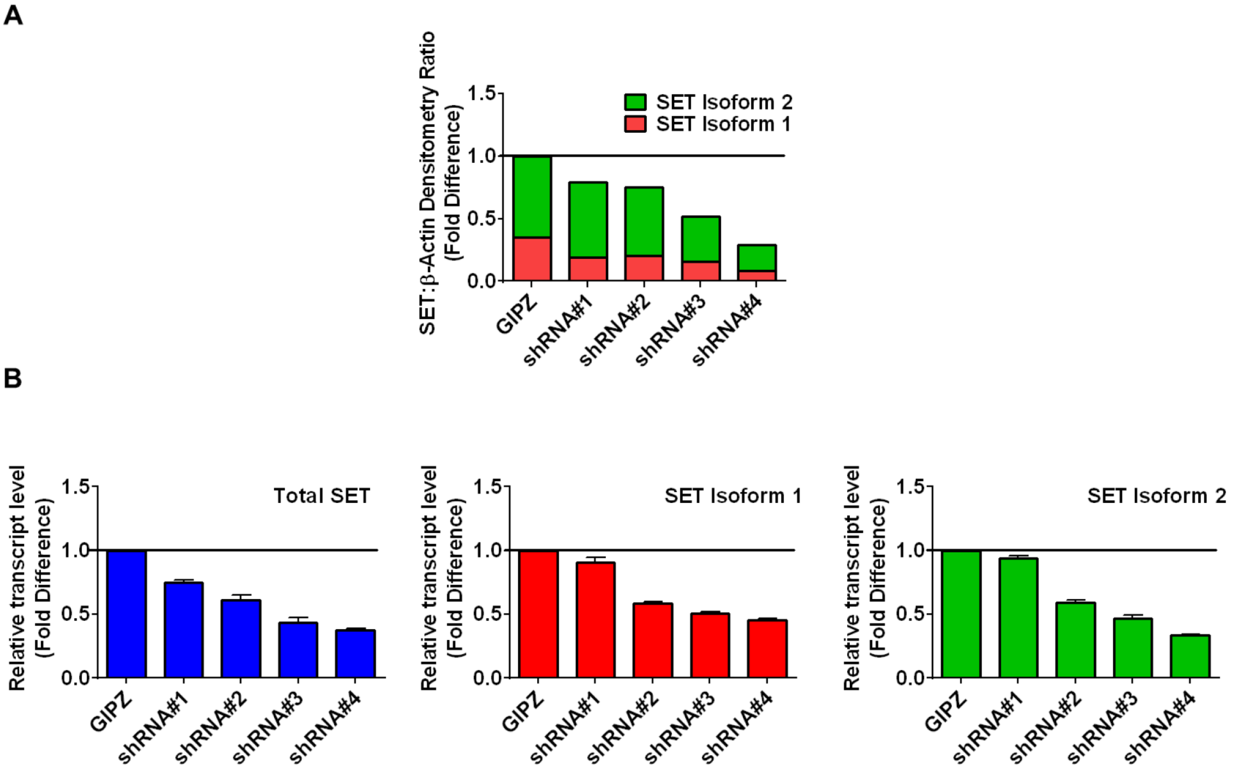

Supplementary Figure 2: Stable knockdown of SET (SET-shRNA) in MIA PaCa-2. (A) Protein levels of SET Isoform 1 and Isoform 2 in SET shRNA (From Fig 3C) transduced MIA PaCa-2 cells were quantified by densitometry and SET:β-actin ratios plotted. **(B)** Expression levels of total SET, isoform 1, and isoform 2 transcripts in MIA PaCa-2 SET-shRNAs analyzed by qRT-PCR with taqman probe. GUSB was used as an internal control. Bars, SD.

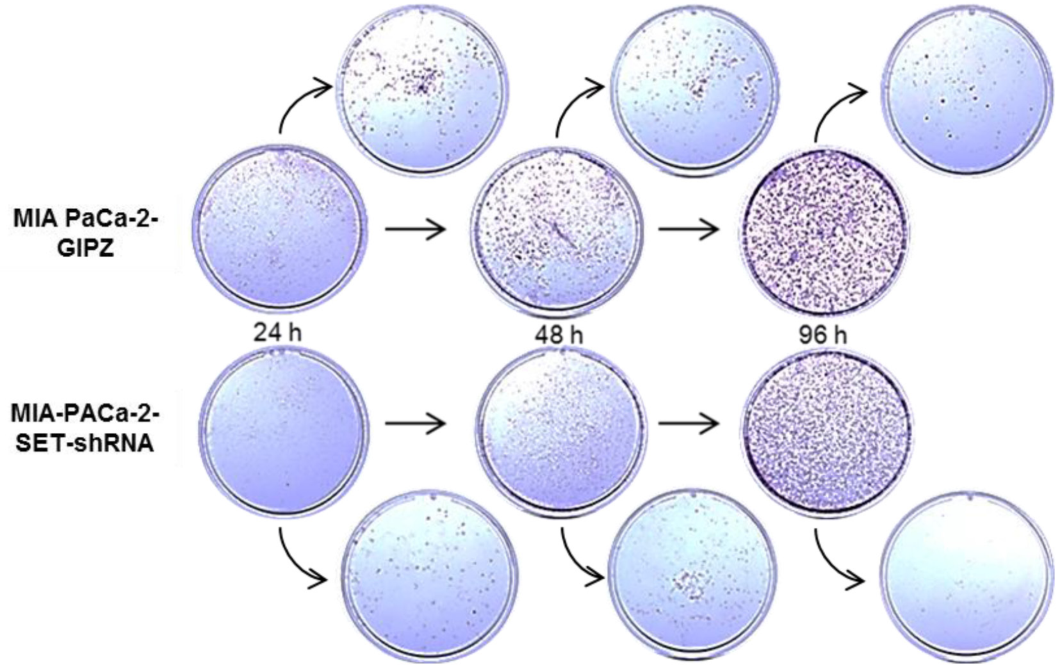

Supplementary Figure 3: Supernatants were collected from cells cultured in primary dishes for the indicated time periods and reseeded and cultured in secondary dishes (curved arrows). After 14 days, colonies developed in secondary dishes were fixed and stained with methylene blue for visualization.

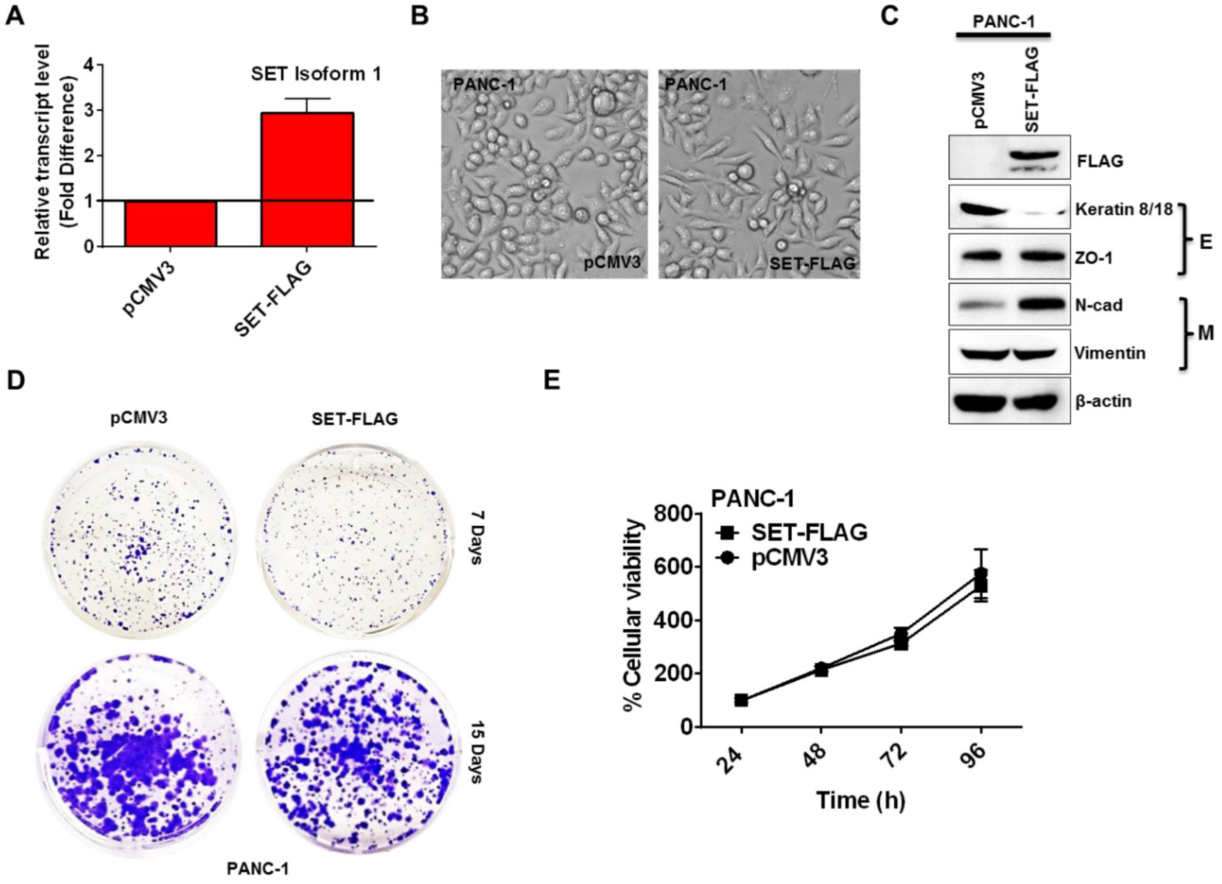

Supplementary Figure 4: SET isoform 1 induces EMT-like characteristics but has no effect on growth of pancreatic cancer cells. **(A)** Stable overexpression of SET Isoform 1 (SET-FLAG) in PANC-1. Expression levels of SET Isoform 1 transcripts in PANC-1 stably overexpressing SET Isoform 1 analyzed by qRT-PCR with taqman probe. GUSB was used as an internal control. **(B-C)** SET isoform 1 (SET-FLAG) overexpression induces EMT like characteristics in PANC-1. **(B)** Cells with overexpression of SET Isoform 1 have a more mesenchymal morphology as compared with control cells (pCMV3). **(C)** Overexpression of SET isoform 1 in PANC-1 increases N-cadherin and decreases Keratin 8/18 protein levels. Whole cell lysates (50 µg) were subjected to Western blotting analysis. β-actin, the internal loading control, is shown with a representative blot. **(D and E)** Expression of SET isoform 1 does not significantly affect the colony formation abilities (D) or the cellular proliferation (E) of PANC-1 unlike SET Isoform 2. For colony formation assays, cells were fixed and stained with crystal violet at indicated time points (D). For cellular viability assays, cells were grown for 96 h and % cellular viability was measured with MTT assay (E). Bars, SD.

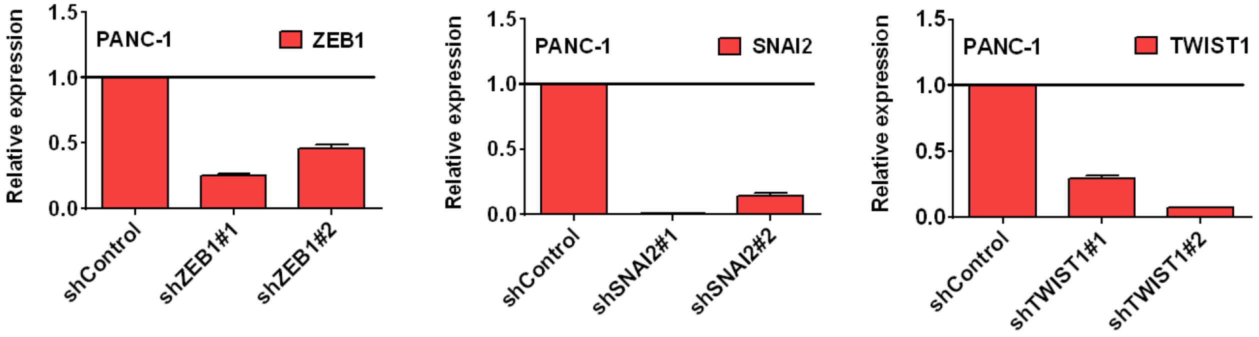

Supplementary Figure 5: Knockdown of EMT-regulating transcription factors (ZEB1, SNAI2, and TWIST1) in PANC-1. Stable knockdown of ZEB1, SNAI2 (Slug), and TWIST1 using respective shRNAs#1-2 in PANC-1. Transcript levels of individual genes analyzed with qRT-PCR and compared against control cells. GUSB was used as an internal control. Bars, SD.

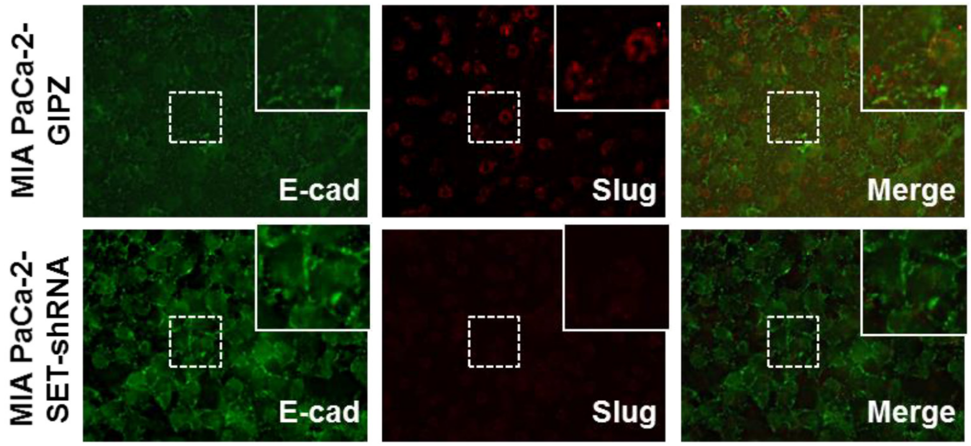

Supplementary Figure 6: E-cadherin and Slug expressions were oppositely correlated in MIA PaCa-2 (GIPZ & SET-shRNA). Slug (red) is expressed in the cytoplasm, while E-cadherin (green) is expressed on the cell surface. E-cadherin images in the left panels (MIA PaCa-2 GIPZ and MIA PaCa-2 SET-shRNA) are also shown in Fig. 4E.

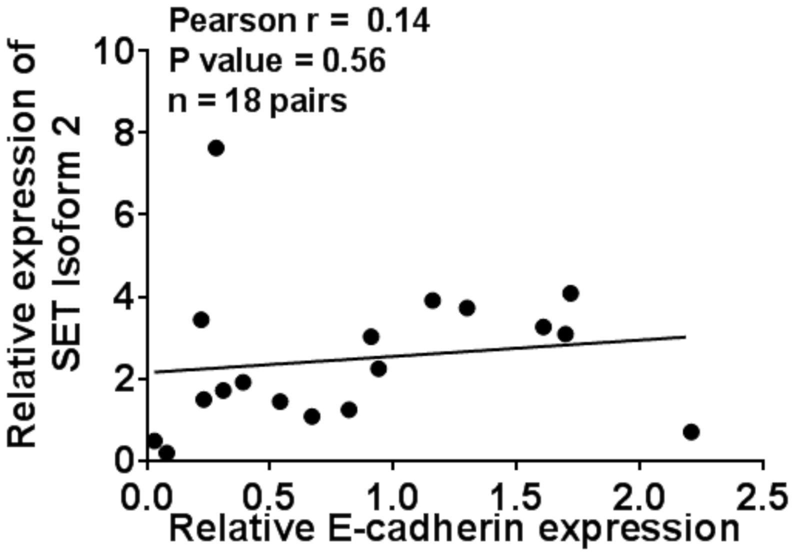

Supplementary Figure 7: E-cadherin transcripts showed no correlation with SET Isoform 2 expression in pancreatic cancer tissues (n = 18).
