# Supplementary material for: SET contributes to the epithelial-mesenchymal transition of pancreatic cancer
Source: Oncotarget. 2017 Jul 7;8(40):67966–79. doi: 10.18632/oncotarget.19067 (PMC5620228; doi:10.18632/oncotarget.19067)
Supplement: Supplementary file 2 [file oncotarget-08-67966-s002.docx]

Supplementary Table 3: List of EMT-related genes (including house-keeping genes) from a RT-PCR profiler array with fold changes on SET overexpression (SET-HA) compared with control cells (pLNCX2) in PANC-1

| **Gene** | **Fold changes** |
| --- | --- |
| B2M | 0.02641744 |
| KRT19 | 0.02815747 |
| RAC1 | 0.03348878 |
| SERPINE1 | 0.05688198 |
| STAT3 | 0.07239985 |
| ITGB1 | 0.12847892 |
| TFPI2 | 0.14260126 |
| SMAD2 | 0.20820298 |
| F11R | 0.28060286 |
| OCLN | 0.29202577 |
| ITGA5 | 0.3041318 |
| TGFB2 | 0.32168345 |
| ITGAV | 0.36714341 |
| JAG1 | 0.39890237 |
| CTNNB1 | 0.50931058 |
| ACTB | 0.54403961 |
| TSPAN13 | 0.55485381 |
| ILK | 0.56211359 |
| PTP4A1 | 0.66357323 |
| EGFR | 0.69161957 |
| TCF4 | 0.75191297 |
| CAV2 | 0.929315 |
| GNG11 | 0.93277537 |
| VPS13A | 1.07857582 |
| SNAI2 | 1.14519684 |
| GSK3B | 1.16586731 |
| MMP2 | 1.17353315 |
| RPLP0 | 1.26119696 |
| DSP | 1.27770484 |
| WNT5B | 1.28951236 |
| AKT1 | 1.29296982 |
| PPPDE2 | 1.31544105 |
| FOXC2 | 1.35636513 |
| PTK2 | 1.4130824 |
| FZD7 | 1.54452754 |
| KRT7 | 1.87084962 |
| AHNAK | 1.98879366 |
| TMEFF1 | 2.09585165 |
| IGFBP4 | 2.23844613 |
| MAP1B | 2.42914667 |
| PLEK2 | 2.55172101 |
| VIM | 2.65347251 |
| TCF3 | 3.06180754 |
| CDH1 | 3.24884654 |
| ZEB1 | 3.25006957 |
| MSN | 3.57283082 |
| SNAI1 | 3.96166146 |
| SPP1 | 3.96816111 |
| CAMK2N1 | 4.28163733 |
| RGS2 | 4.81233008 |
| NOTCH1 | 6.08366874 |
| GAPDH | 6.10897612 |
| COL5A2 | 6.16693483 |
| TGFB1 | 7.34882502 |
| CALD1 | 8.6818085 |
| HPRT1 | 9.03082381 |
| STEAP1 | 9.40652986 |
| BMP1 | 9.96059966 |
| BMP7 | 12.6747694 |
| TMEM132A | 13.4362869 |
| GSC | 14.9391753 |
| ERBB3 | 16.1438257 |
| FN1 | 18.0832565 |
| NUDT13 | 21.0733329 |
| COL3A1 | 27.2239141 |
| MST1R | 29.3503459 |
| SIP1 | 37.3434057 |
| ZEB2 | 42.330646 |
| TIMP1 | 43.5229246 |
| MMP9 | 50.1927679 |
| NODAL | 50.924874 |
| DSC2 | 58.438798 |
| CDH2 | 96.725582 |
| VCAN | 119.71031 |
| MMP3 | 123.933431 |
| BMP2 | 173.029066 |
| COL1A2 | 173.029066 |
| FGFBP1 | 173.029066 |
| IL1RN | 173.029066 |
| SOX10 | 227.98903 |
| ESR1 | 241.349596 |
| SPARC | 266.926106 |
| TWIST1 | 325.540584 |
| WNT11 | 351.069195 |
| KRT14 | 372.559903 |
| SNAI3 | 1138.9965 |
| WNT5A | 3030.90853 |
| PDGFRB | 12133.0749 |
| TGFB3 | 20411.486 |
|  |  |
